# Supplementary material for: A cross-border seroprevalence study on HBV, HCV, HDV and HIV in remote Amazonian communities on the border between French Guiana and Suriname
Source: PLoS One. 2026 May 22;21(5):e0344187. doi: 10.1371/journal.pone.0344187 (PMC13196985; doi:10.1371/journal.pone.0344187)
Supplement: S2 File — (DOCX) [file pone.0344187.s002.docx]

Inclusivity in global research

PLOS’ policy on inclusivity in global research aims to improve transparency in the reporting of research performed outside of researchers’ own country or community and ensures that PLOS publications reporting global research adhere to high standards for research ethics and authorship. Authors of relevant research articles may be asked to complete the questionnaire below, which outlines ethical, cultural, and scientific considerations specific to inclusivity in global research. This questionnaire may be requested when researchers have travelled to a different country to conduct research, if research uses samples collected in another country, research with Indigenous populations or their lands, or if research is on cultural artefacts. Researchers travelling to another country solely to use laboratory equipment will not normally be required to complete the questionnaire. However, the questionnaire can be requested at the journal’s discretion for any submission – if you have been requested to complete this questionnaire by the PLOS journal you submitted to, please do so.

Please complete the questionnaire below and include this as a Supporting Information file with your manuscript. Note that if your paper is accepted for publication, this checklist will be published with your article in the supporting information files. Please ensure that you reference the checklist in the main body of your manuscript. We suggest adding a subsection ‘Inclusivity in global research’ to your Methods section and adding the following sentence: “Additional information regarding the ethical, cultural, and scientific considerations specific to inclusivity in global research is included in the Supporting Information (SX Checklist)”

The questions have been designed to be applicable to a wide range of study types, and there are subsections for both human subjects research and non-human subjects research. If any of the questions are not relevant to your research please mark them as “N/A” as appropriate.

**Ethical considerations, permits and authorship**

*This section is applicable to all research types.*

Provide details as to who granted permissions and/or consent for the study to take place in the Methods section of your manuscript. This should include the names of **all** ethics boards, governmental organizations, community leaders or other bodies that provided approval for the study. If individuals provided approval refer to these people by their role or title but do not list their name(s).

Reported on page number 12 “Ethical and regulatory aspects”.

Patient enrolment took place in both participating countries (French Guiana and Suriname), with no cross-border inclusion: in each country, participants, regardless of country of residence, were enrolled exclusively by local research teams operating on their own territory.

Biological analyses were centralized in Montpellier (France) for reproducibility purposes. This organization was detailed in the protocol submitted to the ethics committees and in the participant information sheet, specifying the transport and storage procedures for DBS samples from each country, including refrigerated transport on dry ice and temporary storage at −20°C in Cayenne. Samples were partially retained locally for biobanking purposes, with no planned genetic analyses, while the remaining portions were sent to the UMR1058 laboratory in Montpellier for hepatitis B, C, D, and HIV testing.

Regulatory procedures were conducted separately in each country according to national regulations, based on a uniform protocol translated and submitted to the relevant authorities. In French Guiana, approval was obtained from the Ethics Evaluation Committee of the National Institute of Health and Medical Research (IRB00003888), and in Suriname from the Ethics Committee of the Ministry of Health (VG 023-16). Additional authorizations were required in French Guiana, including approvals from the Comité de Protection des Personnes Ile de France XI (IDRCB No. 2017-A00200-53), CODECOH (No. DC-2019-3851) (CNIL (No. 16-792), and CCTIRS (No. DR-2017-222). Finally, civil liability insurance covering all participants was secured (No. 01012848-14004).

In addition, permission to conduct the study was granted by the traditional authorities of each participating Indigenous community (Maroon and Amerindian). Depending on the community, approval was provided by the traditional chief (Gran Man), who is the recognized customary leader and may be based in either French Guiana or Suriname. Whenever feasible, these approvals were discussed and obtained during joint meetings involving the traditional authorities and representatives of the research teams from both territories.

Detailed description of the backgound, preliminary field work for study adaptation, interactions with traditional and local authorities, and communication, and logistics of the MaHeVi study, are available in Schaub et al. 2023 [1].

1. Schaub R, Ottevanger MSMD, Harkisoen S, Pesna B, Duijves C, Heemskerk M, et al. Involving hard-to-reach populations is pivotal for the tailoring and implementation of an epidemiological study in cross-border communities of French Guiana and Suriname. Frontiers in Public Health [Internet]. 2023 [cited 2023 Jun 5];11. Available from: https://www.frontiersin.org/articles/10.3389/fpubh.2023.1162705

If there were any deviations from the study protocol after approval was obtained please provide details of these changes in the Methods section of your manuscript.

Reported on page number: not applicable, there has been no deviations from the study protocol.

Did this study involve local collaborators that are residents of the country where the research was conducted or members of the community studied? If you do not have any authors from said communities, please provide an explanation for this below.

This study was conducted by a balanced and equitable partnership of local researchers and research stakeholders based in the territories where the research took place: French Guiana and Suriname. French Guiana, although an overseas territory of France, is geographically and culturally part of the Amazonian region of South America, and the research team was grounded in this local context:

- The study sponsor was Cayenne Hospital, located in French Guiana. One principal investigator was designated in each territory (Mathieu Nacher, French Guiana; Stephen Vreden, Suriname), along with one coordinating investigator per site (Roxane Schaub, French Guiana; Sigrid MacDonald, Suriname).
- Field teams included physicians and nurses residing and practising in the study territories (Stella Hoang, Barbara Biche – French Guiana; Anfernee Neus, Rikesh Bisnajak, Antoon Grunberg, Soeradj Harkinsoen – Suriname), ensuring care delivery and data collection were embedded within local health systems.
- The remaining authors include locally based clinicians specialised in viral hepatitis, health centre managers, and a statistician, all residing in French Guiana or Suriname and actively involved in the regional healthcare and research landscape (Stephen Vreden, Sigrid MacDonald, Soeradj Harkisoen, Rikesh Bisnajak, Richard Naldjinan, Céline Michaud, Mélanie Gaillet, Aude Lucarelli, Cyril Rousseau, Julie Blanc).

Only the authors contributing specifically to laboratory-based analyses were not residents of French Guiana or Suriname (Amandine Pisoni, Karine Bolloré, Janke Schinkel, Emmanuel Gordien, Maria Prins, Edouard Tuaillon).

This study meaningfully involved local collaborators who were both residents of and originating from the communities studied. Community mediators from the Maroon and Amerindian populations were recruited directly within the study sites and worked as key partners in the research process, ensuring culturally safe practices, facilitating trust-building, communication, and locally grounded participant engagement. Their involvement reflects our commitment to inclusive, community-centred and equitable research practices. While their contributions were essential to the successful implementation of the study, they did not fulfil the internationally recognised authorship criteria (ICMJE) for co-authorship. In recognition of their critical role and in line with principles of equitable collaboration and respect for local knowledge, their contributions have been explicitly acknowledged in the Acknowledgements section of the manuscript.

Everyone listed as an author should meet PLOS’ criteria for authorship and all individuals who meet these criteria should be included in the author byline, rather than the acknowledgements. For further information please see the journal’s Authorship Policy.

**Human subjects research (e.g. health research, medical research, cross-cultural psychology)**

Did you obtain written informed consent from a representative of the local community or region before the research took place? How did you establish who speaks for the community? Details of written informed consent obtained from study participants should be reported separately in the Methods section of your manuscript.

Prior to the start of the study, we sought and obtained the approval of the local community authorities (Maroon and Amerindian). Due to the oral tradition of these communities, consent was obtained orally from the recognized community leaders (*capitaines*, *Gran Man* [local titles]), who are regarded as legitimate representatives for decision-making within their communities. Written consent was therefore not obtained at the community level. Details of written informed consent obtained from individual study participants are reported separately in the Methods section of the manuscript (page number 12 “Ethical and regulatory apects”).

How did members of the local community provide input on the aims of the research investigation, its methodology, and its anticipated outcome(s)?

Members of the local communities (Maroon and Amerindian) were actively engaged in methodological aspects of the study, including advising on culturally appropriate modes of communication, participant engagement strategies, and the practical organization of fieldwork. In fact we expanded the region for including participants per special request/demand of the *Gran Man.* In addition, input was sought from local health actors, including healthcare providers and some traditional practitioners, to ensure the study design was aligned with local healthcare practices and community expectations. While the overall research topic—viral hepatitis—was determined by the scope of the funded grant, the contributions of community members and local health actors were essential for adapting the study procedures to the local context and ensuring meaningful and ethical participation.

When engaging with the local community, how did you ensure that the informed consent documents and other materials could be understood by local stakeholders?

Engagement with local stakeholders and the entourage of the traditional chiefs, including older community members who were less likely to be proficient in the official languages, was conducted in the language of their choice. Most stakeholders were proficient in the official language of their territory (French in French Guiana, Dutch in Suriname), but interpreters were provided whenever needed to ensure clear communication and mutual understanding. This approach recognized and respected local governance structures and the authority of the chiefs’ representatives, allowing all participants to contribute meaningfully to discussions on study methodology and implementation while ensuring that language barriers did not limit their participation.

Will the findings of the research be made available in an understandable format to stakeholders in the community where the study was conducted (e.g. via a presentation, summary report, copies of publications, etc.)? Please provide details of how this will be achieved.

Yes, the findings will be shared with stakeholders in the communities where the research was conducted. We will prepare a lay summary of the results, translated into the relevant local languages (French, Dutch, Portuguese, and the main Indigenous languages). The summary will be presented during future visits to the region, using accessible formats such as printed copies, presentations, or posters, and communicated by local team members. This approach ensures that results are understandable, culturally appropriate, and reach both community representatives and local health actors.

**Non-human subjects research using specimens/ animals collected as part of the study, or those housed in archival collections. Examples include archaeology, paleontology, botany and zoology.**

Did the permission you obtained from a local authority to perform the study include an agreement on access to outputs and benefit sharing? This may include procedures to enable fair distribution of the benefits and resources arising from the research performed. Please include any details of Prior Informed Consent and Benefit Sharing Agreements obtained. These may be required by field-specific regulations, for example the Convention on Biological Diversity (CBD) and the associated Nagoya Protocol.

Not applicable (no non-human research)

If the material used in your study was imported, please A) provide the year it was imported and B) indicate whether permits were obtained to import/export the materials used, C) provide details of any permits obtained. If this information is not available, please indicate this.

Not applicable (no non-human research)

If you used archival specimens, please state how the material used in your study was acquired by the institute it is held in and provide details of any permits obtained for the original excavations/ sample collection. If this information is not available, please indicate this.

Not applicable (no non-human research)

How was the potential cultural significance of the materials collected in your study to local communities considered in your research design? Were Indigenous peoples and/or local researchers and institutions involved with archaeological excavations / collection of specimens? If so, please provide a description of their involvement.

Not applicable (no non-human research)

If your manuscript includes photographs of human remains please indicate whether authors obtained permission from descendants or affiliated cultural communities to do so.

Not applicable (no non-human research)
