# Supplementary material for: A cross-border seroprevalence study on HBV, HCV, HDV and HIV in remote Amazonian communities on the border between French Guiana and Suriname
Source: PLoS One. 2026 May 22;21(5):e0344187. doi: 10.1371/journal.pone.0344187 (PMC13196985; doi:10.1371/journal.pone.0344187)
Supplement: Attachment — Submitted filename: Comments.docx [file pone.0344187.s003.docx]

**Comments**

1. Could you clarify the rationale behind offering an "opt-out" for HIV testing, rather than an "opt-in"?
2. Did the demographic characteristics of the 6% who opted out differ from those who consented, and how might this have affected the final reported HIV prevalence?
3. How was the question about "transactional sex" phrased by the cultural mediators to distinguish it from formally declared "sex work"? Understanding this operational definition is crucial for interpreting its significance as a risk factor.
4. The supporting information notes that some initial positive results from Dried Blood Spots were later found to be negative on whole blood samples. Were the final prevalence figures and risk factor analyses based on the initial screening results or only on the confirmed cases?
5. Could you specify the source and year of the census data used for the post-stratification weighting? Given the high mobility in this border region, how accurate and current is this data considered to be?
6. The literature review is well-focused on the Guiana Shield and South America. However, the discussion could be strengthened by contextualizing the findings with recent literature from other low- and middle-income regions facing similar public health challenges. For example, comparing prevalence rates in key populations (like pregnant women) or vaccination barriers could highlight universal themes. The following recent articles, while geographically distant, explore relevant topics and could be considered for inclusion:
   - Seroprevalence of hepatitis B, hepatitis C, and HIV in pregnant women attending a tertiary care hospital in Mogadishu, Somalia, 2017–2021 (DOI: 10.1186/s12879-025-11268-9)
   - Prevalence and associated factors for hepatitis B infection among pregnant women attending antenatal clinic at SOS Hospital in Mogadishu, Somalia (DOI: 10.3389/fgwh.2024.1279088)
   - Uptake of hepatitis B vaccination and associated factors among health sciences students, Mogadishu, Somalia (DOI: 10.3389/fpubh.2023.1203519)
