# Supplementary material for: A cross-border seroprevalence study on HBV, HCV, HDV and HIV in remote Amazonian communities on the border between French Guiana and Suriname
Source: PLoS One. 2026 May 22;21(5):e0344187. doi: 10.1371/journal.pone.0344187 (PMC13196985; doi:10.1371/journal.pone.0344187)
Supplement: Attachment — Submitted filename: To PLOSE ONE.docx [file pone.0344187.s004.docx]

**Date: July 20, 2025**

**To PLOSE ONE**

**Subject: Peer review**

Dear Editor,

I am thankful for the invitation to review a manuscript entitled “cross-border seroprevalence study on HBV, HCV, HDV and HIV in remote Amazonian communities on the border between French Guiana and Suriname.” The topic is potentially good and of global health importance. However, addressing the following comments will enrich the paper further, and my decision is major revision.

**Comments**

**Abstract**

**Background**: The background statements in the abstract part lacks the local and global health importance of dealing with these viruses and why the study is designed trans border.

**Methods**: The methods section lack information about the laboratory methods used to detect these viruses.

The study period is between January 2018 and February 2019. If this were a prospective study, how will the findings represent the current situation? Adding data of recent years can enrich the paper and fill the time gap.

**Key words**: the name of the viruses should fully expressed. Eg. Hepatitis B virus instead of Hepatic B

**Methods**

1. The methods section lacks description of the study area/setting, source and study populations in separate headings.
2. The is no clearly stated exclusion criteria
3. Before the Testing methods and laboratory analyses section, there should be a description of the study variables (dependent and independent variables), sample size determination and sampling techniques, and data collection procedures in a separate headings
4. The methodology also lacks information about data quality assurance measures take.

**Results**

1. The presentation of the results have some redundancy. After dealing with the sociodemographic characteristic, the next sections should be about the prevalence and then the determinants of infection. Hence, table 2 can be addressed in table 3.

**Discussion**

1.	Lastly, the discussion part should include limitations of the study if available.

**Conclusion**

1. The paper lacks conclusion part before the acknowledgement. The authors should draw at least a paragraph about the major conclusions and possible recommendations.
