# Supplementary material for: A cross-border seroprevalence study on HBV, HCV, HDV and HIV in remote Amazonian communities on the border between French Guiana and Suriname
Source: PLoS One. 2026 May 22;21(5):e0344187. doi: 10.1371/journal.pone.0344187 (PMC13196985; doi:10.1371/journal.pone.0344187)
Supplement: Attachment — Submitted filename: 2026.01.14 Response to reviewers.docx [file pone.0344187.s006.docx]

**Authors’ Revisions and Responses to Reviewers’ Comments**

**First revision:**

*We thank the editor and reviewers for their thoughtful and constructive comments. We have carefully revised the manuscript accordingly. Below, we provide a point-by-point response to each comment. Before addressing these comments, we would first like to highlight several changes made to the manuscript that are indirectly related to the reviewer’s comments and important to make following a careful review of the biological results and, in particular, the thresholds used to interpret these results.*

*We had initially set lower thresholds for the detection of HBsAg and anti-HCV antibodies in order to account for the potential lack of sensitivity of DBS compared to serum from venous blood and to minimize the risk of false negatives, which would have been detrimental to the interests of the participants.*

*However, for HBsAg, the results that we were subsequently able to verify on venous blood were consistent with the thresholds provided by the manufacturer for the LIAISON® XL Murex HBsAg Quant kit (DiaSorin). We have therefore considered the thresholds for HBsAg as follows, in accordance with the recommendations of the manufacturers of the kits used: reactive (positive) result if index value ≥1.00, non-reactive (negative) result if index value <1.00.*

*Accordingly, for anti-HCV antibodies, we revised the initially broader cutoffs to bring them closer to the manufacturer’s recommendations for the LIAISON® XL MUREX HCV Ab assay (DiaSorin): non-reactive if S/CO <0.80, equivocal if 0.80–1.00, and reactive if ≥1.00. However, because the assay was performed on DBS eluates, we retained a slightly broader equivocal range, classifying results between >0.20 and <1.00 as equivocal.*

*This has changed the number of participants in the “HBsAg+” and “HCV+” columns in Tables 1 and 2. We have therefore modified the corresponding numbers in the manuscript text as well as in Additional Tables 2 and 3 (where the modified elements are highlighted in yellow).*

*Overall, we reviewed all serological cutoff values used in the study and clarified the laboratory procedures in the revised manuscript, specifically in the Materials and Methods section. In addition, detailed information on assay thresholds has been added to Supplementary Table 2.*

**Reviewer #1:**

**The authors performed a seroprevalence study of hepatitis B, C and D and HIV, in the border between French Guiana and Suriname. Some concerns need to be addressed before acceptance of this manuscript.**

*We sincerely thank reviewer #1 for their insightful comments and questions, which we will attempt to address below point by point.*

**1. The results show an apparent low prevalence of HBsAg in the Amerindian ethnic group. This may be due to the low number of individuals from this ethnic group, in addition to the age of the participants: higher HBsAg prevalence may have been found at lower ages. It would be interesting to include in Table 1 anti-HBc prevalence and perform the analysis of risk factors for HBV according to this marker and not (only) to HBsAg. The presence of anti-HBc is only presented in a supplemental table.**

*Notably, the vaccination uptake in the Amerindian group was significantly higher than in the non-Amerindian group. Consequently, in the first paragraph of the results, we removed results that were redundant with Table 1 and replaced them with results comparing the sociodemographic characteristics (age, sex, education) of vaccinated and unvaccinated participants.*

*We agree with the reviewer to include risk factors for HBV exposure based on anti-HBc seropositivity (regardless of HBsAg status) and have added these description to Tables 1 and 2. As the reviewer correctly noted, exploring HBV infection risk factors only in patients with acute or chronic infection (based on HBsAg positivity) was not the most appropriate, as it does not consider infected individuals who have cleared the infection - a significant proportion of those exposed to the virus, particularly adults [*[*Hepatitis B WHO fact sheets*](https://www.who.int/news-room/fact-sheets/detail/hepatitis-b)*]. We therefore revised the HBV risk factor analyses, initially performed only on individuals with positive HBsAg, with an analysis comparing individuals who were HBV exposed to HBV susceptible individuals, which is not only more relevant from a disease history perspective, but also allows us to present much more robust models, due to the larger number of events.*

**2. Additional Table 2. Footnote is incomplete: some superscript symbols (*, for example), are not explained in the footnote.**

*We thank the reviewer for their vigilance. We have completed the footnotes for Additional Table 2, corrected “HBe Ab”, which was a typo, to “HBe Ag”, and made changes to the numbers in some cells (highlighted in yellow) following the redefinition of certain thresholds as explained at the beginning of this document:*

- *we initially indicated “‡ 5/52 positive were later tested on whole blood samples and confirmed negative”, but we removed this sentence because these 5 participants had in fact negative HBs Ag with the new threshold definition we describe at the beginning of the document, and replaced with the following precision “‡ 7/46 positive were later tested on whole blood samples and confirmed positive”*
- *“† 3/17 [Anti-HCV Ab] positive were later tested on whole blood samples and confirmed negative” was changed to “4 [Anti-HCV Ab] positives were not retested and 1 was already known and treated”*
- *“* 3/9 equivocal anti-HCV Ab were later tested on whole blood samples and confirmed negative” was changed to “* 4/12 equivocal anti-HCV Ab were later tested on whole blood samples and confirmed negative”.*

**3. According to Additional Table 2, the prevalence of anti-HBc is indeed quite high, not reflected in the prevalence of HBsAg. It is important to analyze this (high) prevalence of exposure in each population group and to correlate with the risk factors. This is even more important than the correlation with HBsAg.**

*Please see our response to comment 1. We agree with the reviewer and, in addition to modifying the analysis of HBV infection risk factors to include not only HBsAg-positive participants but also anti-HBc-positive participants, we also performed subgroup analyses by sex and by language community, the detailed results of which have been added in Additional Tables 4 and 5, respectively.*

**4. Did the authors consider to analyze occult HBV infection in at least part of the anti-HBc positive samples?**

*This is a very valid point. However, occult hepatitis B infection (OBI) is defined by the persistence of HBV DNA in the absence of detectable HBsAg. By nature, the viral load is extremely low, usually below 200 IU/mL, and often at the level of 10–50 IU/mL, sometimes detectable only intermittently with highly sensitive PCR assays.*

*Given that the threshold for detection of HBV DNA assays performed on dried blood spots (DBS) is approximately 50-fold higher than that of plasma-based molecular methods, the probability of detecting such low and fluctuating levels of viremia becomes negligible. As a result, DBS testing is not appropriate for the diagnosis or monitoring of OBI, since it would systematically underestimate the true prevalence,*

*Therefore, HBV DNA testing on DBS cannot be recommended for the evaluation of occult hepatitis B, as it lacks the analytical sensitivity required to capture the very low viral loads that define this condition. Furthermore, we know that anti-HBs is less sensitive on DBS, therefore per WHO recommendations we have included only anti-HB-core positive results as part of resolved HBV infections.*

*It is not appropriate for diagnosis or monitoring of occult HBV infections (OBI; defined by persistence of HBV DNA in the absence of HBsAg) since HBV loads in case of OBI are generally extremely low, and not detected by molecular DBS testing. Hence, we may have missed OBI.*

*However, we have added OBI as a limitation to the manuscript: “Fifth, as low level HBV DNA is difficult to detect using DBS, we were unable to collect data on occult HBV infection - defined as presence of HBV DNA in the liver with detectable or undetectable HBV DNA levels in serum, in individuals who are HBsAg seronegative) (*[*EASL 2017 Guidelines*](https://easl.eu/wp-content/uploads/2018/10/HepB-English-report.pdf)*). In terms of public health, and prevention of HBV transmission, this aspect warrants further research”.*

**5. Another concern is the presence of false positive results in these population groups. In rural populations, the presence of polyclonal activation might be frequent, due for example to parasitic infections. This may lead to false positive antibody results, less expected for anti-HBc, which is an inhibition assay (then more specific) but relatively frequent with anti-HCV in some population groups (see for example Rogers KJ, et al., J Clin Lab Anal. 2023 Apr;37(7):e24887. doi: 10.1002/jcla.24887. Epub 2023 Apr 27). The presence of only 20% of viremic samples among the anti-HCV positive ones suggest that some false positive results may have occurred. The authors may consider a second testing with another commercial assay or applying a more stringent criteria of positivity based on signal to cutoff ratio.**

*We thank reviewer 1 for this comment, which lead us to carefully review our biological results and, in particular, the thresholds used to interpret these results.*

*As indicated in the introduction to this letter, we had initially set lower thresholds for the detection of anti-HCV antibodies in order to account for the lower sensitivity of DBS compared to serum from venous blood. We were subsequently able to verify on venous blood a few equivocal anti-HCV Ab participant’s result, and they were all confirmed negative. For anti-HCV Ab, we revised the initially broader cutoffs to bring them closer to those indicated by the manufacturer of the LIAISON® XL MUREX HCV Ab kit (DiaSorin): non-reactive (negative) if S/CO value <0.80; equivocal if S/CO value [0.80-1.00], reactive (positive) if S/CO value ≥1.00. After re‑analyzing the results, we identified five anti‑HCV positive cases, four of whom also tested positive for HCV RNA which is more consistent with expected HCV serology.*

**6. Discussion. The comparison between anti-HBc prevalence should be included, and limitation on anti-HCV testing.**

*We have now added the discussion on anti-HBcore seropositivy. Due to the anti-HCV results being in line with expected HCV serology, we no longer view the anti-HCV testing using DBS as a limitation, and did not add this.*

**Reviewer #2:**

*We would like to thank the reviewer for their insightful comments and questions, which we will attempt to address below point by point.*

**1. Could you clarify the rationale behind offering an "opt-out" for HIV testing, rather than an "opt-in"?**

*We chose opt-out regimens rather than opt-in for HIV testing, because opt-out can substantially increase HIV testing compared to opt-in schemes (Montoy JCC, Dow WH, Kaplan BC. Patient choice in opt-in, active choice, and opt-out HIV screening: randomized clinical trial. BMJ. 2016 Jan 19;352:h6895; doi:* [*https://doi.org/10.1136/bmj.h6895*](https://doi.org/10.1136/bmj.h6895)*).*

*We have added this clarification and the associated reference to the manuscript by replacing the following sentence: “This opt-out was offered because of our perception that HIV testing might be a factor in not participating.” with* *“We chose the opt-out option for HIV testing based on data indicating this may increase test acceptance* *rate [ref].”*

**2. Did the demographic characteristics of the 6% who opted out differ from those who consented, and how might this have affected the final reported HIV prevalence?**

*We compared the demographic characteristics of the 134 participants who refused the HIV test with the 2152 participants who accepted it. There were no differences in sex, age, professional activity, but there were differences for the following characteristics:*

*- country of birth: there were less participants who refused HIV testing among those born in Suriname (4.2%) compared to participants born in French Guiana (8.8%) or in another country (5.3%), p=0.002.*

*- country of residence: there were less participants who refused HIV testing among those living in Suriname (3.1%) compared to participants living in French Guiana (7.5%) or in another country (6.7%), p=0.002.*

*- school level: HIV testing refusal rate in participants who have been to high school was higher (11.2%) compared to participants who have either never been to school / primary / middle school and higher education, with HIV testing refusal rates of 4.1, 3.8, 6.0 and 6.6%, respectively, p<0.0001.*

*We have included the following sentence to the manuscript in the results section:*

- *Six percent (n=134) participants opted out HIV-testing. There were significant differences in country of birth and residence, and educational level between participants who opted out for HIV testing compared to those that did not. There were no differences in sex, age, work, or other determinants.*

*And in the discussion/limitation section:*

- *Indeed, 6% opted out for HIV-testing and some of them did so declaring they were HIV-positive. However, the opt-out policy presumably led to a high participation rate; the HBV and HCV prevalence is however, likely more accurate than for HIV.* *Despite the opt-out, we believe that due to the large sample size, the HIV prevalence estimates are robust.*

**3. How was the question about "transactional sex" phrased by the cultural mediators to distinguish it from formally declared "sex work"? Understanding this operational definition is crucial for interpreting its significance as a risk factor.**

*The specific question that the mediators asked was “The last time you had sex, did you pay your partner or were you paid by your partner?”. This precision appears in our manuscript as the following footnote in Table 2: “Transactional sex was defined as having paid for or having been paid in exchange for sex”. This formulation implied that we were asking specifically about money and not gifts, compared to what has been done by other authors, for example* [*Ranganathan et al*](https://onlinelibrary.wiley.com/doi/10.7448/IAS.19.1.20749)*. in young women in rural South Africa, where they distinguished receiving money and gifts in 2 separate questions.*

*In our study, because of the design (same questionnaire for both women and men) and setting, we preferred to use a single succinct question, so as not to overload the questionnaire, which could have slowed down inclusions and discouraged some people from participating.*

*Moreover, this questionnaire was developed based on an extensive preliminary assessment in the communities, including focus group discussions and interviews with key individuals. We believe that the questionnaire was therefore tailored to the population.*

*However, distinguishing sex work and transactional sex, as well as the nuance between money and gifts is interesting, and it might be worthwhile to explore these issues in a more focused project on the Maroni river, especially among girls and young women.*

**4. The supporting information notes that some initial positive results from Dried Blood Spots were later found to be negative on whole blood samples. Were the final prevalence figures and risk factor analyses based on the initial screening results or only on the confirmed cases?**

*As indicated in the introduction to this letter, we had initially set lower thresholds for the detection of HBsAg and anti-HCV antibodies than the thresholds provided by the manufacturer for the LIAISON® XL Murex HBsAg Quant kit (DiaSorin) in order to account for the potential lack of sensitivity of DBS compared to serum from venous blood and to minimize the risk of false negatives, which would have been detrimental to the interests of the participants.*

*However, for HBsAg, the results that we were subsequently able to verify on venous blood were consistent with the thresholds provided by DiaSorin. We have therefore considered the thresholds for HBsAg as follows, in accordance with the recommendations of the manufacturers of the kits used: reactive (positive) result if index value ≥1.00, non-reactive (negative) result if index value <1.00.*

*For anti-HCV Ab, the manufacturer of the LIAISON® XL MUREX HCV Ab kit (DiaSorin) indicate the following thresholds: non-reactive (negative) if S/CO value <0.80; equivocal if S/CO value [0.80-1.00], reactive (positive) if S/CO value ≥1.00. We decided to apply a slightly broader definition for equivocal results, keeping results between > 0.20 and <1 in this category.*

*Overall, we reviewed all serological cut-off values used in the study and clarified the laboratory procedures in the revised manuscript, specifically in the Materials and Methods section. In addition, detailed information on assay thresholds has been added to Supplementary Table 2.*

*This has changed the number of participants in the “HBsAg+” and “HCV+” columns in Tables 1 and 2. We have therefore modified the corresponding numbers in the manuscript text (track changes), as well as in Additional Tables 2 and 3 (where the modified elements are highlighted in yellow).*

*In conclusion all prevalences were calculated on the updated screening results, using the manufacturers thresholds for HBsAg and anti-HCV Ab positivity.*

**5. Could you specify the source and year of the census data used for the post-stratification weighting? Given the high mobility in this border region, how accurate and current is this data considered to be?**

*The data used for post-stratification weighting for French Guiana are population as of January 1st, 2018, obtained from the national census on the website of INSEE (National Institute of Statistics and Economic Studies), with data available by age group and gender for each municipality available via the following links:*	[*https://www.insee.fr/fr/statistiques/5397445?sommaire=5397467&geo=COM-97353*](https://www.insee.fr/fr/statistiques/5397445?sommaire=5397467&geo=COM-97353)*;*	[*https://www.insee.fr/fr/statistiques/5397445?geo=COM-97362*](https://www.insee.fr/fr/statistiques/5397445?geo=COM-97362)*;*	[*https://www.insee.fr/fr/statistiques/5397445?geo=COM-97357*](https://www.insee.fr/fr/statistiques/5397445?geo=COM-97357)*;*	[*https://www.insee.fr/fr/statistiques/5397445?geo=COM-97360*](https://www.insee.fr/fr/statistiques/5397445?geo=COM-97360)*.*

*For Suriname, the data used for post-stratification weighting are resorts’ population obtained from the 2012 national census, with data available by age group and gender for each resort (the resort being the smallest available geographical unit in the census, just below the district geographical unit). These data are available on* [*https://statistics-suriname.org/wp-content/uploads/2019/05/Publicatie-Census-8-Volume-1-Demografische-en-Sociale-Karakteristieken-en-Migratie.pdf*](https://statistics-suriname.org/wp-content/uploads/2019/05/Publicatie-Census-8-Volume-1-Demografische-en-Sociale-Karakteristieken-en-Migratie.pdf)*.*

*We clarified this in the manuscript by replacing the sentence* *“Post-stratification weighting was carried out on the population structure in terms of age and sex of each municipality, based on census data from Suriname and French Guiana in each municipality” with “Post-stratification weighting was carried out on the population structure in terms of age and sex of each geographical unit, based on 2012 census data from Suriname (for each resort) and 2018 census data for French Guiana (for each municipality)”.*

*Regarding the accuracy of the data used, we based our calculations on data from the 2018 census for French Guiana, and for the last census conducted in Suriname in 2012 (there has been no other census conducted since). The data published subsequently for 2018 are only estimates and are not broken down by resort or even by district (*[*https://statistics-suriname.org/wp-content/uploads/2023/03/DEMOGRAFISCHE-DATA_DEMOGRAPHIC-DATA-2018-2021-februari-2023-1.pdf*](https://statistics-suriname.org/wp-content/uploads/2023/03/DEMOGRAFISCHE-DATA_DEMOGRAPHIC-DATA-2018-2021-februari-2023-1.pdf)*), and it appears that at the national level, the age and sex structure in the 2018 estimates has changed very little compared to 2012. We therefore prefer to use the detailed resort data from the 2012 census rather than the national estimates for 2018.*

**6. The literature review is well-focused on the Guiana Shield and South America. However, the discussion could be strengthened by contextualizing the findings with recent literature from other low- and middle-income regions facing similar public health challenges. For example, comparing prevalence rates in key populations (like pregnant women) or vaccination barriers could highlight universal themes. The following recent articles, while geographically distant, explore relevant topics and could be considered for inclusion:**

**o Seroprevalence of hepatitis B, hepatitis C, and HIV in pregnant women attending a tertiary care hospital in Mogadishu, Somalia, 2017–2021 (DOI: 10.1186/s12879-025-11268-9)**

**o Prevalence and associated factors for hepatitis B infection among pregnant women attending antenatal clinic at SOS Hospital in Mogadishu, Somalia (DOI: 10.3389/fgwh.2024.1279088)**

**o Uptake of hepatitis B vaccination and associated factors among health sciences students, Mogadishu, Somalia (DOI: 10.3389/fpubh.2023.1203519)**

*We agree with the reviewer’s suggestion. Comparing our data to other middle-income countries with similar HBV, HCV and HIV modes of transmission is indeed interesting. However, we had already surpassed the number of references in our manuscript. We have, however, added one of the suggested references given the similarity in population and relevance to the study.*

**Reviewer#3**

*We would like to thank the reviewer for their insightful comments and questions, which we will attempt to address below point by point.*

**Dear Editor,**

**I am thankful for the invitation to review a manuscript entitled “cross-border seroprevalence study on HBV, HCV, HDV and HIV in remote Amazonian communities on the border between French Guiana and Suriname.” The topic is potentially good and of global health importance. However, addressing the following comments will enrich the paper further, and my decision is major revision.**

**Comments**

**Abstract**

**Background: The background statements in the abstract part lacks the local and global health importance of dealing with these viruses and why the study is designed trans border.**

*We thank the reviewer for this helpful comment. In response, we have revised the background section of the abstract to better emphasize both the local and global health importance of addressing viral hepatitis and HIV, as well as the rationale for our trans‑border study design. Specifically, we added the following sentence:* “National epidemiological data are essential to achieve viral hepatitis elimination goals. However, viral hepatitis and HIV epidemiology in French Guiana and Suriname is mainly limited to urban areas.

**Methods: The methods section lack information about the laboratory methods used to detect these viruses.**

*We thank the reviewer for this comment. We included more detailed laboratory methods in the abstract as follows: “Participants were tested for viral hepatitis and HIV using serological and molecular analyses: HBV (HBsAg, anti-HBcore antibodies, anti-HBs, HBV DNA), HCV (HCV-antibodies, HCV RNA), HDV (HDV-antibodies), and HIV (HIV-antibodies/antigen, HIV RNA).”.*

**The study period is between January 2018 and February 2019. If this were a prospective study, how will the findings represent the current situation? Adding data of recent years can enrich the paper and fill the time gap.**

*We thank the reviewer for this important comment. We agree that including more recent data would enrich the paper and provide additional insight into the current situation. However, given the remote context in which the study was conducted, obtaining these data was already extremely challenging and resource‑intensive, and redeploying a similar organization at present would not be feasible. We nevertheless believe that the findings remain highly relevant, as they provide rare epidemiological information from hard‑to‑serve populations and highlight structural barriers to testing and treatment that are unlikely to have changed substantially in recent years.*

***Key words: the name of the viruses should fully expressed. Eg. Hepatitis B virus instead of Hepatic B***

*We agree that fully expressing the names of the viruses improves clarity, exposure, and representation. Accordingly, we have revised the keywords as follows:* Hepatitis B virus, Hepatitis C virus, HIV, Hepatitis D virus, rural communities, Indigenous communities, tribal communities, hard‑to‑serve populations, epidemiology, determinants, knowledge, attitudes, practices, and beliefs.

**Methods**

**The methods section lacks description of the study area/setting, source and study populations in separate headings.**

*We thank the reviewer for this valuable comment. For more clarity, we added “and population” to the “Study design” subheading*. *Given the complexity of the Maroni area and the significant efforts made prior to the MaHeVi study itself, we chose to describe the MaHeVi study setting, source of study population and important details in a separate publication, which we cite in this part of the Methods section (Reference #20). In this article, we extensively describe the involved populations, their lifestyles, the awareness and communication and anthropological studies ahead of the project, as well as the MaHeVi study communication, design and conduct and their implications.*

**The is no clearly stated exclusion criteria**

*We thank the reviewer for this observation. To clarify, we have now added the following sentence in the Methods section:* “No further exclusion criteria were applied beyond the inclusion criteria specified above.”

**Before the Testing methods and laboratory analyses section, there should be a description of the study variables (dependent and independent variables), sample size determination and sampling techniques, and data collection procedures in a separate headings**

**The methodology also lacks information about data quality assurance measures take.**

*We thank the reviewer for this valuable comment. In response, we have revised and expanded the Methods section.*

*We have described all the variables, in brief, in the methods section under determinants of HBV, HCV, and HIV infection, and KAP-B regarding HBV and HCV infection. Sample size determination has been described later on in the methods section, and data collection was described in a separate publication. Furthermore, we expanded the data analysis paragraph.*

*We compared participant characteristics across HBV exposure groups (acute or chronic infection, or resolved infection), acute or chronic HCV infection, and HIV infection. Risk-factor analyses focused on HBV-exposed versus HBV-susceptible individuals, and HIV-positive versus HIV-negative individuals; determinants of HCV infection were not evaluated due to the very small number of HCV cases. Odds ratios (ORs) and 95% CIs were computed using bivariable logistic regression, and linearity of continuous variables was assessed.*

**Results**

**The presentation of the results have some redundancy. After dealing with the sociodemographic characteristic, the next sections should be about the prevalence and then the determinants of infection. Hence, table 2 can be addressed in table 3.**

*We thank the reviewer for this helpful comment. In response, we have reanalyzed the data and updated the tables and text to reduce redundancy and improve clarity. In addition to analyses of chronic or acute HBV infection, we now present risk factor analyses based on anti‑HBc seropositivity (regardless of HBsAg status), which have been incorporated into Tables 1 and 2. Accordingly, the HBV risk factor analyses, previously limited to individuals with positive HBsAg, were revised to compare HBV‑exposed individuals with HBV‑susceptible individuals. This approach is more relevant from a disease history perspective and provides more robust models due to the larger number of events.*

**Discussion**

**Lastly, the discussion part should include limitations of the study if available.**

*We thank the reviewer for this comment. The limitations of the study were already included in the Discussion section; however, to improve clarity and ensure they are more explicitly presented, we have updated and expanded this section in the revised manuscript. We have also included the limitation of possibly missing occult HBV infection in our study population as follows.* *“Fifth, as low level HBV DNA is difficult to detect using DBS, we were unable to collect data on occult HBV infection - defined as presence of HBV DNA in the liver with detectable or undetectable HBV DNA levels in serum, in individuals who are HBsAg seronegative [51]**. In terms of public health, and prevention of HBV transmission, this aspect warrants further research.”*

**Conclusion**

**The paper lacks conclusion part before the acknowledgement. The authors should draw at least a paragraph about the major conclusions and possible recommendations.**

*While our final paragraph already included the major conclusions and recommendations, we recognize that this may not have been sufficiently clear. We have therefore updated and expanded this paragraph to explicitly present the study’s key conclusions and recommendations before the acknowledgements section.*

**Second revision:**

*We thank the editor and the reviewers for their careful re-evaluation of our manuscript and for the positive assessment of the revisions made.*

**Response to Reviewer#3 :**

*As suggested, we have revised the manuscript to clearly distinguish the Conclusion from the Discussion. A standalone Conclusion section has now been created at the end of the main text. In addition, the Conclusion is now explicitly identified as a separate component in the Abstract, in accordance with the reviewer’s recommendation.*

*These changes are purely structural and do not affect the scientific content or interpretation of the results.*
